# Fabrication of CS/GA/RGO/Pd composite hydrogels for highly efficient catalytic reduction of organic pollutants

**DOI:** 10.1039/d0ra01884h

**Published:** 2020-04-16

**Authors:** Lei Ge, Meng Zhang, Ran Wang, Na Li, Lexin Zhang, Shufeng Liu, Tifeng Jiao

**Affiliations:** Pollution Prevention Biotechnology Laboratory of Hebei Province, School of Environmental Science and Engineering, Hebei University of Science and Technology Shijiazhuang 050018 P. R. China; State Key Laboratory of Metastable Materials Science and Technology, Yanshan University Qinhuangdao 066004 P. R. China tfjiao@ysu.edu.cn; Key Laboratory of Optic-electric Sensing and Analytical Chemistry for Life Science, Ministry of Education, College of Chemistry and Molecular Engineering, Qingdao University of Science and Technology Qingdao 266042 P. R. China

## Abstract

In this study, natural polymer material chitosan (CS) and graphene oxide (GO) with large specific surface area were used to prepare a new CS/RGO-based composite hydrogel by using glutaraldehyde (GA) as cross-linking agent. In addition, a CS/GA/RGO/Pd composite hydrogel was prepared by loading palladium nanoparticles (Pd NPs). The morphologies and microstructures of the prepared hydrogels were characterized by SEM, TEM, XRD, TG, and BET. The catalytic performance of the CS/GA/RGO/Pd composite hydrogel was analyzed, and the experimental results showed that the CS/GA/RGO/Pd composite hydrogel had good catalytic performance for degradation of *p*-nitrophenol (4-NP) and *o*-nitroaniline (2-NA). Therefore, this study has potential application prospect in wastewater treatment and provides new information for composite hydrogel design.

## Abstract 2

New functional CS/GA/RGO/Pd composite hydrogels are prepared *via* a self-assembly process, demonstrating potential applications in catalysis as well as composite materials.

## Graphical abstract

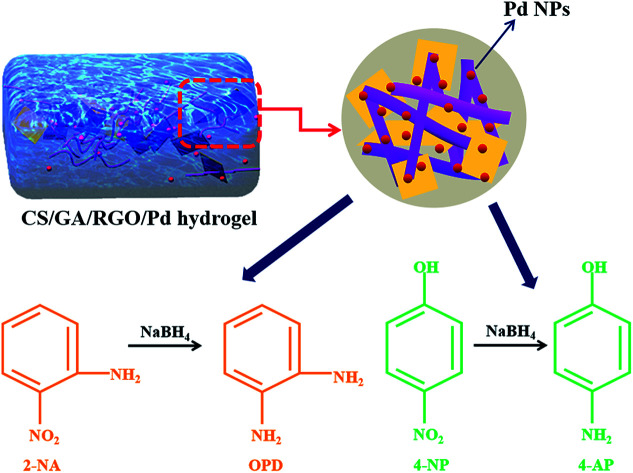

## 1 Introduction

In recent years, nitrobenzene-based water pollutants such as *p*-nitrophenol (4-NP) and *o*-nitroaniline (2-NA) have become an urgent problem to be solved, including being toxic and difficult to degrade.^1–5^ Targeting these organic substances, precious metal catalysts are mainly used to react with reducing agents to generate compounds that are less toxic, easily degradable, and less polluting to water, or non-polluting, thereby achieving the reuse of water resources.^5,6^ Precious metal nanoparticles have special optical, catalytic, electrochemical and mechanical properties, so they have potential applications in optics,^7–9^ catalysis,^10–12^ microelectronics,^13–15^ biomedicine^16,17^ and many other fields. The most common used catalysts are gold,^18–20^ silver^1,21–24^ and palladium.^25–27^ In this work, palladium nanoparticles (Pd NPs) were used to catalyze the reaction of 4-NP and 2-NA with sodium borohydride (NaBH_4_).

The preparation of polysaccharide hydrogel is based on the cross-linking of polysaccharide chains through chemical binding,^28,29^ which gives the hydrogels porous network properties, swelling and flexibility.^30^ Moreover, such hydrogels have a large number of hydroxyl and carboxyl groups, rich functional groups, biocompatibility and biodegradability.^31^ In recent years, they have been extensively studied and designed as wastewater treatment materials. In this experiment, biodegradable natural polymer material chitosan (CS) is a green and pollution-free functional material with simple source and low cost.^32,33^ Moreover, CS containing hydroxyl and amino groups is a good candidate for pollutant removal^34,35^ and can be used as an ideal material for hydrogel preparation.^36^ In addition, CS molecular skeleton contains a certain number of polar functional groups (such as amino and hydroxyl groups), and these groups can strongly chelate with transition metals, which make CS can be used as heterogeneous carrier for catalytic applications in organic synthesis.^37–39^ At present, CS has been widely used as a transition metal catalyst carrier.^40,41^ However, the preparation of hydrogels by CS alone cannot achieve a good catalytic effect. Graphene oxide (GO) composites have excellent properties such as high hydrophilicity, good dispersibility, large specific surface area and strong ion exchange capacity.^42–45^ By compounding CS with GO can enhance the stability of CS hydrogel and provide a higher specific surface area. At the same time, higher specific surface area provides favorable conditions for loading metal nanoparticles.^46^

In addition, in order to better follow the concept of green chemistry, ascorbic acid was selected as the reducing agent in this experiment.^47–49^ Ascorbic acid acts as an eco-friendly medium reducing agent can reduce GO into reduced graphene oxide (RGO) and to form RGO hydrogel.^50,51^ At the same time, the presence of ascorbic acid will also reduce the metal ions to metal elements *in situ*. The reduction of oxygen-containing functional groups on the surface after reduction of GO to RGO provides more available space for the fixation of Pd NPs. Therefore, the nanoparticles can be tightly fixed on the surface of RGO without aggregation. In addition, RGO can lead to the improvement of electron mobility due to its unique properties, thus promoting the reduction performance, which is conducive to the catalytic reduction of nitrobenzene pollutants in water.^52^

## 2 Materials and methods

### 2.1 Materials

Chitosan (CS, >90% degree of deacetylation) was purchased from Sinopharm Chemical Reagent Co., Ltd (Beijing, China). Sodium borohydride (NaBH_4_), *p*-nitrophenol (4-NP) and *o*-nitroaniline (2-NA) were purchased from Alfa Aesar (Tianjin, China) chemical. Other materials, such as graphite powder (C, 99.85% purity), potassium permanganate (KMnO_4_), potassium nitrate (KNO_3_), hydrogen peroxide (H_2_O_2_, 30%, w/w), glutaraldehyde (analytically pure, GA, 50%), palladium chloride (PdCl_2_), ascorbic acid, sulfuric acid (H_2_SO_4_, 98%), acetic acid (analytically pure, CH_3_COOH, >99.5%) and hydrochloric acid (HCl), were obtained from Aladdin Chemicals (Tianjin, China) without further purification. The water used in all the experiments was obtained using a Milli-Q ultrapure water purification system.

### 2.2 Preparation of CS/GA/RGO hydrogel

The modified Hummers' method was used to extract GO from graphite powder,^53^ and the obtained brown-yellow solution was dialysis and freeze-dried to obtain the GO sample, which was then prepared into 5 mg mL^−1^ GO dispersion by ultrasonic dispersion. Prepare 10 mL of 3% (v/v) glacial CH_3_COOH solution, 500 mg of CS was put into the CH_3_COOH solution, and dissolve by ultrasound to obtain 5 mg mL^−1^ CS solution. Prepare 2 mL 1% GA solution, 1 mL PdCl_2_ solution (10 mg mL^−1^), 2 mL ascorbic acid solution (100 mg mL^−1^) for use. 1 mL ascorbic acid solution (100 mg mL^−1^) was added into 4 mL GO dispersion (5 mg mL^−1^) and then stirred for 0.5 h in a water bath at 40 °C. Then add 4 mL CS solution (5 mg mL^−1^) to the beaker under stirring continuously. After that, 1 mL of 1% GA solution was added to the beaker and the obtained mixture was stirred for 1 h at 60 °C to obtain CS/GA/RGO hydrogel. The obtained sample was dialyzed for three days.

### 2.3 Preparation of CS/GA/RGO/Pd hydrogel

1 mL ascorbic acid solution (100 mg mL^−1^) was added into 4 mL GO dispersion (5 mg mL^−1^) and stirred in a water bath at 40 °C for 0.5 h. Then added 0.6 mL PdCl_2_ solution (10 mg mL^−1^) and 1 mL ascorbic acid solution (100 mg mL^−1^) to the beaker, the mixture was heated to 90 °C in a water bath with stirring for 1 h. Then 4 mL CS solution (5 mg mL^−1^) was added into the beaker, kept stirring, and finally added 1 mL of 1% GA solution to the beaker. The water bath was heated to 60 °C and stirred for 0.5 h to obtain CS/GA/RGO/Pd hydrogel. The prepared CS/GA/RGO/Pd hydrogel was dialyzed for three days.

### 2.4 Catalytic experimental tests

The catalytic performance of the composite hydrogel was mainly evaluated by the degradation of the mixed solution of NaBH_4_ and 4-NP or 2-NA. The prepared CS/GA RGO/Pd composite hydrogel was mixed with 2-NA aqueous solution (1 mL, 5 mM) or 4-NP (1 mL, 5 mM), and then fresh NaBH_4_ solution (10 mL, 0.01 M) was added. Take the supernatant at a certain time interval to measure the UV spectrum by UV-visible spectrophotometer, which can be used to determine the catalytic performance of the composite hydrogel. In order to examine the reusability of the catalyst, the CS/GA RGO/Pd hydrogel catalyst was collected from the reaction solution, washed three times with ethanol, froze drying and employed for the subsequent cycle of the catalysis experiment.^54^

### 2.5 Characterization

The GO sheets and xerogels used in the present study were obtained using a lyophilizer at −50 °C with a FD-1C-50 lyophilizer instrument from Beijing Boyikang Experimental Instrument Co., Ltd. (Beijing, China) to completely remove water over 2–3 days. The nanostructures of all the lyophilized samples were studied by a field-emission scanning electron microscope (SEM) (S-4800II, Hitachi, Japan) with 5–30 kV accelerating voltage, and transmission electron microscopy (TEM) with a 20 kV accelerating voltage (HT7700, Hitachi High-Technologies Corporation, Japan). X-ray diffraction (XRD) was measured on an X-ray diffractometer equipped with a Cu Kα X-ray radiation source and a Bragg diffraction setup (SMART LAB, Rigaku, Japan). Thermogravimetry (TG) was conducted by using a NETZSCH STA 409 PC Luxxsi multaneous thermal analyzer (Netzsch Instruments Manufacturing Co., Ltd, Germany) in argon gas. The specific surface area and pore diameter distribution of the hydrogels were determined by Brunauer–Emmett–Teller measurement (BET) (ASAP 2460) in a N_2_ atmosphere. The UV-Vis spectra was recorded on an UV-TU1 810PC spectrophotometer.

## 3 Results and discussion

### 3.1 Characterization and analysis of hydrogels

**Fig. 1:**
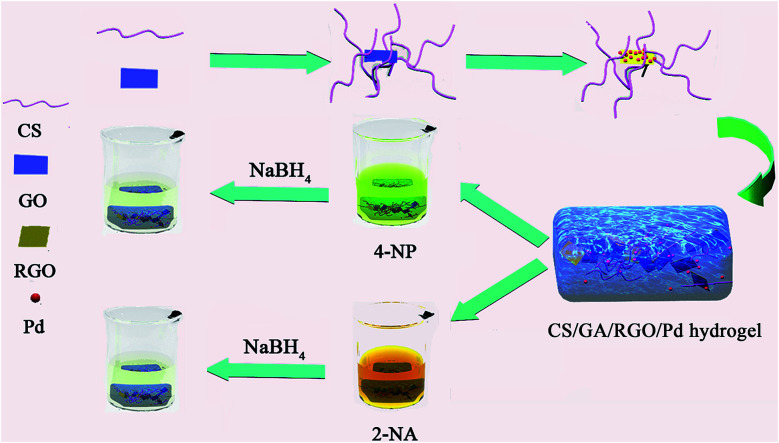
Preparation and catalytic process of CS/GA/RGO/Pd hydrogel.

**Fig. 2:**
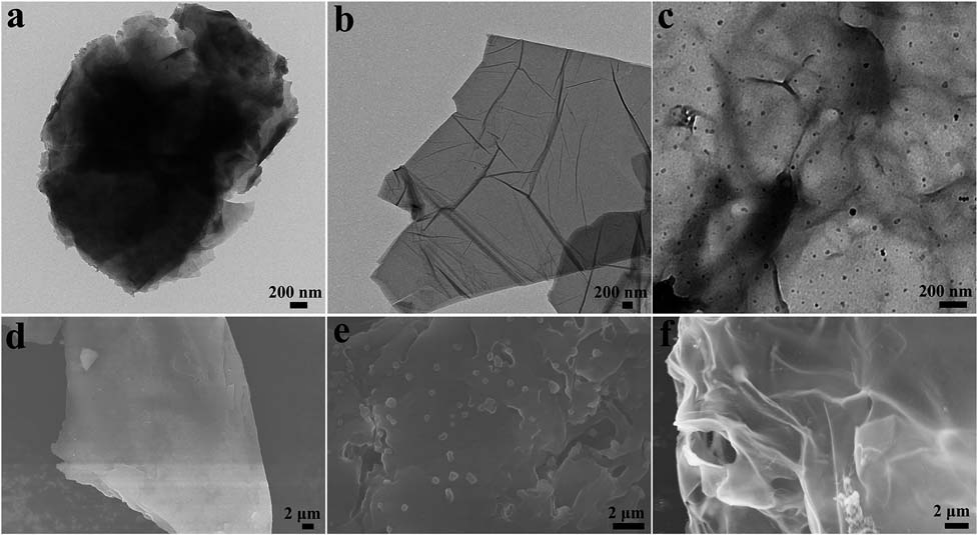
TEM images of CS/GA/RGO hydrogel (a), GO (b), CS/GA/RGO/Pd hydrogel (c); SEM images of CS/GA/RGO hydrogel (d), CS/GA/RGO/Pd hydrogel (e), CS (f).

The preparation and catalytic process of CS/GA/RGO/Pd hydrogel are shown in Fig. 1. The CS/GA/RGO/Pd composite hydrogel was prepared by CS and GO self-assembly, GA as crosslinking agent and ascorbic acid as reducing agent. The prepared hydrogel was utilized as catalysts and reaction media for the reduction reactions of 2-NA and 4-NP with aqueous NaBH_4_. In order to understand the microstructure of the prepared hydrogel, GO, CS/GA/RGO gel, CS/GA/RGO/Pd gel TEM images and CS, CS/GA/RGO gel, CS/GA/RGO/Pd gel SEM images are shown in Fig. 2. Fig. 2a–c shows the TEM results of CS/GA/RGO hydrogel, GO, CS/GA/RGO/Pd hydrogel. Compared to the TEM image of GO sheet, composite hydrogels appear darker in TEM images. This is due to the self-assembly of GO and CS molecules to form a three-dimensional network structure with thicker layers, as shown in Fig. 2a and c. In addition, Fig. 2c shows that some particles are present in the composite hydrogel, which attributed to Pd NPs. It is worth noting that the diameters of Pd NPs are mainly in the range of 20–50 nm, which can be mainly due to *in situ* reduction of Pd salts in composite hydrogel. The surface of CS/GA/RGO hydrogel (Fig. 2d) is smooth, while CS/GA/RGO/Pd hydrogel (Fig. 2e) surface is loaded with many small particles, indicating that the PdNPs were successfully loaded on the surface of composite hydrogel.

**Fig. 3:**
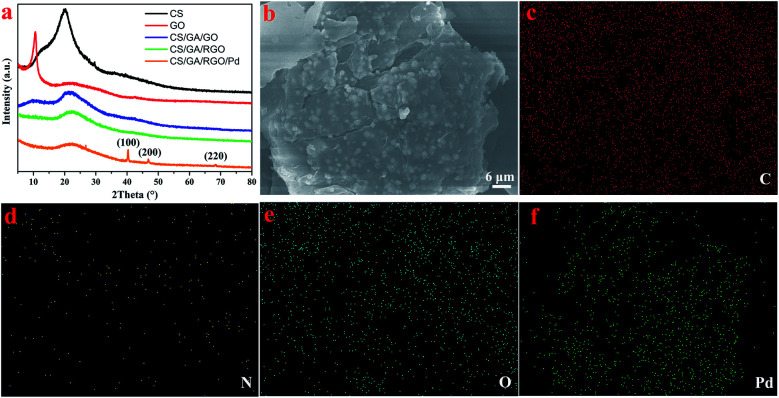
XRD patterns (a) of CS, GO, CS/GA/GO hydrogel, CS/GA/RGO hydrogel, CS/GA/RGO/Pd hydrogel; SEM image (b) and element mappings (c–f) of CS/GA/RGO/Pd hydrogel.

XRD is used to analyze the structure and composition of composite hydrogels, as shown in Fig. 3a. The diffraction peak of graphite is generally around 26°, and it can be seen that the characteristic peak of graphite disappears in the GO XRD spectrum, and a sharp diffraction peak appears at 2*θ* = 10.5°, confirming that graphite is transformed into GO. The diffraction peak of CS/GA/GO hydrogel appeared at 2*θ* = 10.6° and 21.8°, where 2*θ* = 10.6° was consistent with the GO diffraction peak. The diffraction peak of CS/GA/RGO hydrogel appeared at 2*θ* = 22.3°, and there is no characteristic peak around 2*θ* = 10.5°, while the diffraction peak of RGO is generally around 23°,^51,55–57^ so it can be determined that GO has been reduced to RGO. According to the Bragg equation 2*d* sin *θ* = *nλ* (*λ* = 1.54 Å), the crystal surface spacing of CS/GA/RGO is larger than that of CS, which is due to the crosslinking of RGO by CS molecule, increasing the spacing and shifting the peak position. In the XRD pattern of CS/GA/RGO/Pd hydrogel, the diffraction peak appeared at 2*θ* = 22.3°, 40.2°, 46.7°, 68.2°. The diffraction peaks at 40.2°, 46.7° and 68.2° are consistent with the Pd standard card (PDF # 89-4897). The diffraction peak at 2*θ* = 40.2°, 2*θ* = 46.7° and 2*θ* = 68.2° can be respectively assigned to the (111), (200) and (220) crystal plane of Pd, indicating that Pd particles are successfully loaded. In addition, SEM measurement is used to further confirm that the composite hydrogel was successfully loaded with Pd NPs, as shown in Fig. 3b–f. Fig. 3f shows that large amounts of Pd elements are distributed. Combined with XRD characterization, the Pd elementary has been reduced. As an eco-friendly moderate reducing agent, ascorbic acid reduces GO *in situ*, thereby transforming the GO composite hydrogel into a RGO-based hydrogel. At the same time, ascorbic acid reduced Pd^2+^*in situ* to Pd in the hydrogel.

**Fig. 4:**
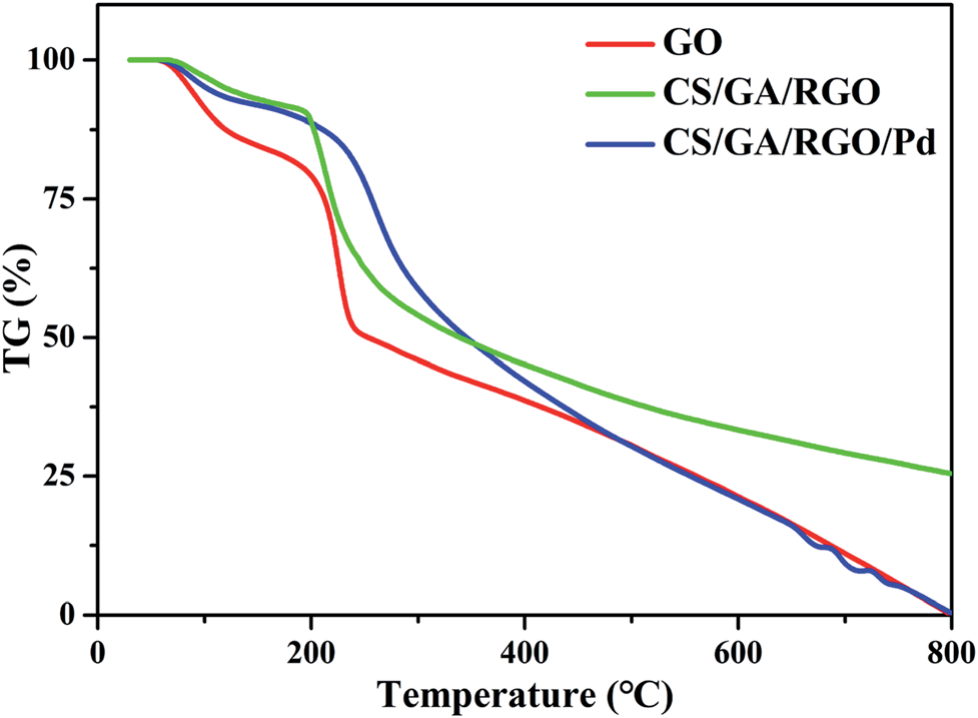
TG curves of GO, CS/GA/RGO hydrogel, CS/GA/RGO/Pd hydrogel.

TG is used to characterize the thermal stability of the prepared samples. TG curves of GO, CS/GA/RGO gel and CS/GA/RGO/Pd gel are performed in an argon atmosphere, as shown in Fig. 4. From the TG curve of GO, it can be observed that 10% of the weight loss occurs near 100 °C, which is obviously caused by the evaporation of water molecules adsorbed on the material. Next, a significant weight loss process of about 30% occurred between 150 °C and 240 °C. This is because as temperature rises and unstable oxygen-containing functional groups undergo pyrolysis at high temperatures, losing small molecules such as CO, CO_2_ and water vapour. When the temperature reaches 240 °C, the thermal weight loss of GO exceeds 50%. The thermal weight loss of CS/GA/RGO/Pd composite hydrogel is only 18% when the temperature reaches 240 °C, which indicates that the unstable oxygen-containing functional groups on the surface of GO are cross linked with CS, making the thermal stability of CS/GA/RGO/Pd hydrogel better than GO. The main thermal weight loss of CS/GA/RGO/Pd hydrogel occurs between 220 °C and 300 °C, which is about 30% weight loss. At 200 °C and 800 °C, CS/GA/RGO/Pd composite hydrogel has the obvious quality loss, which is mainly due to the cracking of the oxygen-containing segments in GO and CS. Compared with CS/GA/RGO/Pd hydrogel, CS/GA/RGO hydrogel decreased faster than CS/GA/RGO/Pd hydrogel between 220 °C and 300 °C, which indicated that the thermal stability of CS/GA/RGO/Pd hydrogel had been greatly improved.

**Fig. 5:**
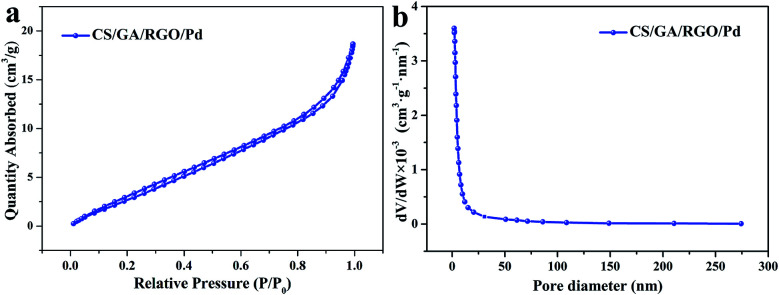
Nitrogen adsorption–desorption isotherms of CS/GA/RGO/Pd hydrogel (a), and pore size distributions of CS/GA/RGO/Pd hydrogel (b).

BET measurement is used as the main way to measure the specific surface area and pore size of a substance, and the results are shown in Fig. 5. Fig. 5a shows the adsorption–desorption isotherm of CS/GA/RGO/Pd hydrogel. It can be seen that in the range of relative pressure from 0 to 1, the adsorption isotherm is a typical type IV adsorption isotherm with a significant lag ring, which belongs to a typical mesoporous adsorption isotherm. The data in Fig. 5a indicate that a large number of microporous and mesoporous structures are present in the CS/GA/RGO/Pd composite hydrogel. Fig. 5b shows that the pore size of the CS/GA/RGO/Pd hydrogel is mainly distributed between 2–10 nm. In addition, the physical characteristics of nitrogen adsorption–desorption of CS/GA/RGO/Pd hydrogel are calculated by BET method, as shown in Table 1. The specific surface area of the hydrogel is 15.5314 m^2^ g^−1^, the average pore size is 7.06935 nm, and the average pore volume is 0.026234 cm^3^ g^−1^.

**Table 1:** Physical data of the obtained xerogels from hydrogels

Sample	Specific surface area (m^2^ g^−1^)	Average pore diameter (nm)	Pore volume (cm^3^ g^−1^)
CS/GA/RGO/Pd gel	15.5314	7.06935	0.026234

The porous structure is important for heterogeneous catalysts so that the reactants could be efficiently contacted with the active sites. The nanoporous structure with high surface area and sufficient space is beneficial to obtain dispersed active sites, which can easily support metal particles and achieve high catalytic performance. As a porous structure material, CS/GA/RGO/Pd hydrogel is beneficial to improve the catalytic performance. Pd NPs can be immobilized on the amino group of CS by chelation.^38,55^ In addition, there is a strong interaction between Pd and the oxides of RGO, and Pd NPs can also be loaded on RGO.^52^ Both CS and RGO can be used as the carrier of catalyst to immobilize Pd NPs, which has synergistic performance and is conducive to the loading of Pd NPs.^55,56^ There are interaction forces between Pd and CS, RGO, so Pd NPs can be loaded on the surface and pores of the hydrogel, providing more space for active sites. The porous structure and high-efficiency loading of CS/GA/RGO/Pd hydrogel greatly increase the catalytic rate.

### 3.2 Catalytic analysis of composite hydrogels

**Fig. 6:**
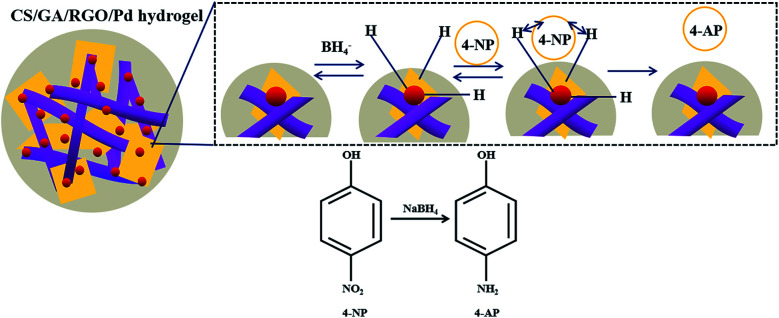
Schematic of the Langmuir–Hinshelwood model for the catalytic reduction of 4-NP.

Fig. 6 shows the mechanism of the catalytic reduction of 4-NP by CS/GA/RGO/Pd hydrogel. The catalytic reduction of 4-NP mainly depends on a process of electron transfer from BH_4_^−^ to receptor 4-NP. In the reaction system, the BH_4_^−^ ion attaches to the surface of the catalyst and then transfers electrons to the surface of the catalyst.^58^ At the same time as electron transfer, hydrogen atoms are generated. The hydrogen atoms attack 4-NP and reduce it, and the 4-NP is reduced to *p*-aminophenol (4-AP).

**Fig. 7:**
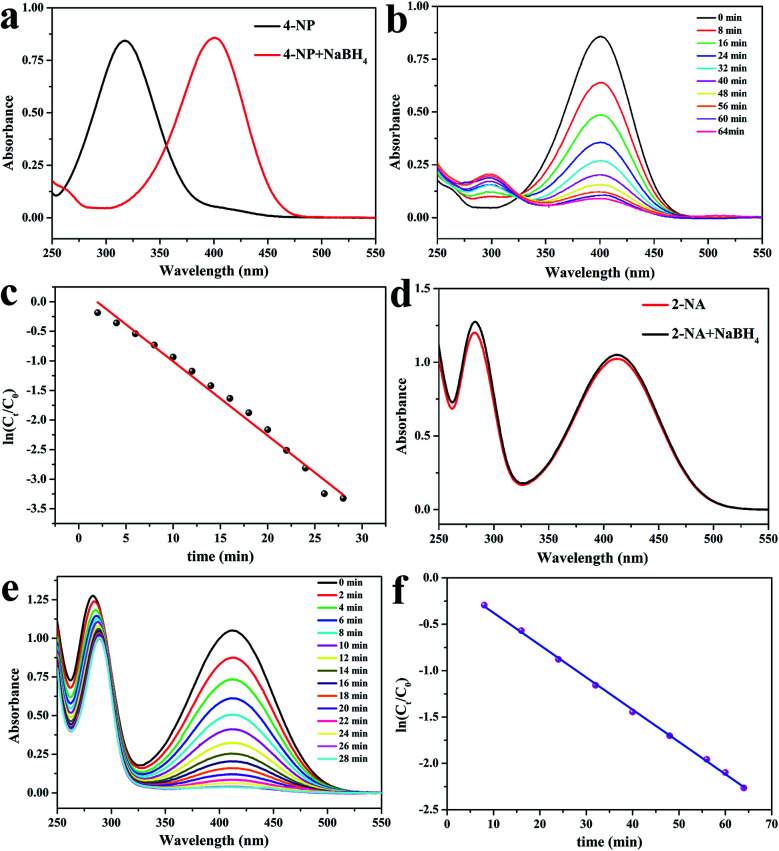
UV-Vis spectra of 4-NP before and after adding NaBH_4_ (a), UV-Vis spectra for the catalytic reduction of 4-NP (b), plots of ln(*C*_*t*_/*C*_0_) *versus t* for the catalytic reduction of 4-NP (c), UV-Vis spectra of 2-NA before and after adding NaBH_4_ (d), UV-Vis spectra for the catalytic reduction of 2-NA (e), plots of ln(*C*_*t*_/*C*_0_) *versus t* for the catalytic reduction of 2-NA (f).

Fig. 7 shows the catalytic performances of CS/GA/RGO/Pd hydrogel for catalyzing 4-NP and 2-NA solutions. Fig. 7a shows the UV absorption spectra of 4-NP solution and 4-NP solution after adding NaBH_4_. It can be seen that the maximum absorption wavelength of 4-NP solution changes from 317 nm to 401 nm after adding NaBH_4_, which due to the addition of NaBH_4_ make 4-NP become *p*-nitrophenol ion, and the corresponding UV characteristic absorption peak shifts to 401 nm. Fig. 7d shows the UV absorption peak of 2-NA and 2-NA solution after adding NaBH_4_. The UV absorption curves of the two solutions are not significantly different. When the catalyst CS/GA/RGO/Pd hydrogel was added, the catalysis experiment officially started, and the ultraviolet absorption spectrum curves during the catalysis process are shown in Fig. 7b and e. Fig. 7b exhibits the UV absorption spectrum of 4-NP catalytic process. The UV absorption spectrum of the reaction solution is measured every certain time (4 min). It can be seen that the characteristic absorption peak (401 nm) of 4-NP does not decrease after 64 min. However, the intensity of the absorption peak (295 nm) of 4-AP increases with the increase of catalytic time and finally does not change, which proves that 4-NP in the solution is completely catalyzed to form 4-AP. Fig. 7e shows the results of catalytic time and UV absorption intensity of 2-NA catalyzed by CS/GA/RGO/Pd composite hydrogel. It can be seen that the characteristic absorption peak (413 nm) corresponding to 2-NA completely disappeared within 28 min, indicating that 2-NA had been completely catalyzed into *o*-phenylenediamine (OPD). The results indicate that the same catalyst has different catalytic efficiency for different substrates.

In order to characterize the catalytic performance of the catalyst, that is, the speed of the catalytic reaction, the data obtained from the catalytic process is fitted. The fitting principle is as follows: since the concentration of NaBH_4_ is in large excess to that of 4-NP (*C*_NaBH_4__/*C*_4-NP_ = 400), the reduction can be considered as a pseudo first-order reaction with regard to 4-NP alone. Therefore, the reaction kinetics can be described as ln(*C*_*t*_/*C*_0_) = −*kt*, where *k* is the apparent first-order rate constant (min^−1^), *t* is the reaction time. *C*_*t*_ and *C*_0_ are the concentrations of 4-NP at time *t* and 0, respectively. According to the Lambert–Beer's law (*A* = *εcl*), it can be seen that the value of concentration *C*_*t*_/*C*_0_ in solution is directly proportional to the value of *A*_*t*_/*A*_0_, where *A*_*t*_ and *A*_0_ are the corresponding absorbance at the wavelength of 401 nm at the time *t* and 0, respectively. The absorbance data at the maximum absorption wavelength were processed by linear fitting, and the relationship between catalytic time *t* and ln(*C*_*t*_/*C*_0_) is shown in Fig. 7c and f. The linear fitting constant of 4-NP and 2-NA is 0.0348 min^−1^ and 0.125 min^−1^, respectively. The larger the linear fitting constant is, the better the catalytic effect is. The data indicating that CS/GA/RGO/Pd hydrogel has better catalytic effect for 2-NA reaction. The comparison of the catalytic activity of CS/GA/RGO/Pd catalyst in the reduction of 4-NP and 2-NA with different reported catalysts is presented in Tables 2 and 3, respectively.^25,59–67^ The results show that CS/GA/RGO/Pd catalyst has good catalytic performance. Moreover, the raw materials and preparation process of CS/GA/RGO/Pd hydrogel are green, pollution-free and environmentally friendly.^68–74^

**Table 2:** Comparative catalytic capacities of relative catalysts for catalytic reduction of 4-NP in reported literatures

Catalyst	Rate constant *k* (min^−1^)	Ref.
Cu/CS-CMM	0.126	59
CS/Pd-0.5%	0.117	60
PEI-Pd0.5	8 × 10^−2^	61
Pd@CH	3.3 × 10^−2^	25
PdNPs-embedded	5.2 × 10^−3^	62
CS/GA/RGO/Pd	3.48 × 10^−2^	This work

**Table 3:** Comparative catalytic capacities of relative catalysts for catalytic reduction of 2-NA in reported literatures

Catalyst	Rate constant *k* (min^−1^)	Ref.
MCA-Pd/Au(1/1)	0.28	63
Pd NPs@Sch-boehmite	0.162	64
MnFe_2_O_4_@SiO_2_@Ag	0.12	65
Ag@CAF	5.3 × 10^−2^	66
AgNPs/RGO	2.6 × 10^−2^	67
CS/GA/RGO/Pd	0.125	This work

**Fig. 8:**
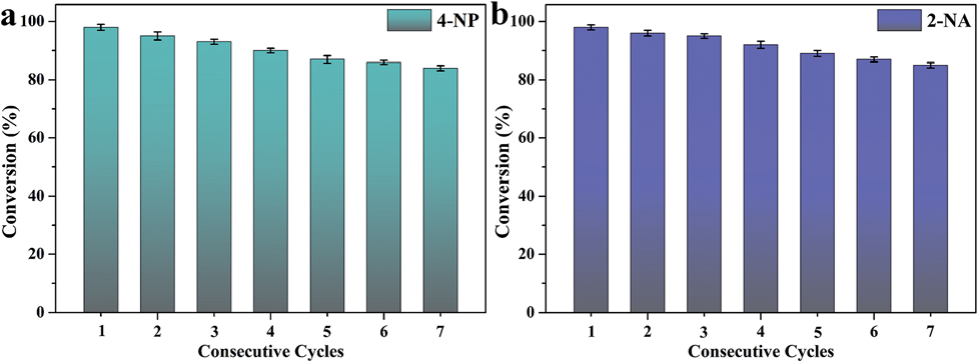
CS/GA/RGO/Pd composite hydrogel catalyzes the stability of 4-NP (a) and 2-NA (b) in different continuous cycles.

After seven catalytic cycles, Fig. 8 shows that the catalytic efficiency of CS/GA/RGO/Pd composite hydrogel for the NaBH_4_ and 4-NP mixture solution. The conversion value could maintain 83%, and the catalytic efficiency for NaBH_4_ and 2-NA mixture solution could maintain 85%. The results of catalytic cycling experiments shows that CS/GA/RGO/Pd composite hydrogel has high catalytic performance and stability, demonstrating broad application prospects in the fields of catalysts and composite materials.^75–86^

## 4 Conclusions

In summary, two kinds of composite hydrogels CS/GA/RGO and CS/GA/RGO/Pd were successfully prepared by self-assembly, and their morphologies and microstructures were characterized. The results show that the prepared hydrogel had porous microstructure, large specific surface area and high thermal stability. The hydrogel network can prevent the aggregation of metal nanoparticles, so CS/GA/RGO hydrogel was used as the carrier for the synthesis of CS/GA/RGO/Pd catalyst in this experiment to increase the contact area between reactants and the catalyst, thus facilitating the catalysis. Pd NPs in CS/GA/RGO/Pd hydrogel was used as catalyst medium for the reduction reaction of 4-NP and 2-NA with aqueous NaBH_4_. The experimental results confirmed that the prepared CS/GA/RGO/Pd composite hydrogel had good catalytic performance for 4-NP and 2-NA. Moreover, the synthesized composite hydrogel exhibited a better catalytic performance for 2-NA. This study has a potential application prospect in the wastewater treatment.

## Conflicts of interest

There are no conflicts to declare.

## Supplementary Material
